# Modeling Cell Selectivity of Antimicrobial Peptides: How Is the Selectivity Influenced by Intracellular Peptide Uptake and Cell Density

**DOI:** 10.3389/fmedt.2021.626481

**Published:** 2021-02-22

**Authors:** Bethany R. Schefter, Shokoofeh Nourbakhsh, Sattar Taheri-Araghi, Bae-Yeun Ha

**Affiliations:** ^1^Department of Physics and Astronomy, University of Waterloo, Waterloo, ON, Canada; ^2^Department of Physics and Astronomy, California State University, Northridge, CA, United States

**Keywords:** antimicrobial peptides, peptide activity and selectivity, biophysical modeling, Langmuir binding model, minimal inhibition concentration, minimal hemolytic concentration

## Abstract

Antimicrobial peptides (AMPs) are known to attack bacteria selectively over their host cells. Many attempts have been made to use them as a template for designing peptide antibiotics for fighting drug-resistant bacteria. A central concept in this endeavor is “peptide selectivity,” which measures the “quality” of peptides. However, the relevance of selectivity measurements has often been obscured by the cell-density dependence of the selectivity. For instance, the selectivity can be overestimated if the cell density is larger for the host cell. Furthermore, recent experimental studies suggest that peptide trapping in target bacteria magnifies the cell-density dependence of peptide activity. Here, we propose a biophysical model for peptide activity and selectivity, which assists with the correct interpretation of selectivity measurements. The resulting model shows how cell density and peptide trapping in cells influence peptide activity and selectivity: while these effects can alter the selectivity by more than an order of magnitude, peptide trapping works in favor of host cells at high host-cell densities. It can be used to correct selectivity overestimates.

## Introduction

Antimicrobial peptides (AMPs) are naturally-occurring peptide antibiotics and attack bacteria selectively over host cells (1–3). AMPs are mostly cationic and have stronger binding affinity for bacterial membranes, which carry a large fraction of anionic lipids (1–4). Their amphiphilic structure enables them to attach to and perturb membranes (1–5). While membrane perturbation is not the sole mechanism of action, it is the first decisive event they induce (1, 2, 5). Indeed, AMPs are multitasking molecules: they are pore formers, metabolic inhibitors (1, 2), and/or immunomodulators (6–8). Their membrane-perturbing ability has, however, spurred many attempts to use them as a template for designing potent peptide antibiotics, especially for fighting conventional drug-resistant bacteria (1, 2, 4, 9). Developing bacterial resistance against membrane-perturbing peptides would involve “costly” redesigning of their membranes (1). Nevertheless, pathogens can evolve antimicrobial resistance (10, 11). Consequences of this need to be considered in our endeavor in searching for potent peptide antibiotics. Despite this challenge, the therapeutic potential of these multitasking molecules has generated interest in designing optimized peptides [see a recent review (7) and references therein].

A central concept in assessing peptide potency is “peptide selectivity.” For a given peptide, it is quantified by the ratio of a minimum hemolytic concentration (MHC) to a minimum inhibitory concentration (MIC) [see for instance; (9)]. For large MHC/MIC, there is a range of peptide concentration at which a given peptide is active against bacteria only. The requirement of a minimum peptide concentration (either MIC or MHC) for membrane rupture suggests that cell density is a control parameter for peptide activity and selectivity, as recently discussed (12, 13). Increasing the cell density is equivalent to reducing the amount of peptides available to each cell. As a result, MICs and MHCs increase as the cell density increases; the ratio MHC/MIC is cell-density dependent.

A related quantity is a threshold coverage of peptides on membranes (3, 14–17). Let *P*/*L* be the molar ratio of bound peptides to lipids. At the MIC or MHC, *P*/*L* reaches the threshold value, *P*/L*, beyond which bound peptides permeabilize the membrane. The value of *P*/L* depends on the type of peptide and lipid (3, 14–17). It is typically larger for lipid membranes mimicking bacterial membranes.

The correct interpretation of selectivity measurements has often been obscured by the cell-density dependence of the selectivity (12, 13, 18). For instance, the selectivity can be overestimated if the cell density is larger for the host cell. Furthermore, a number of recent studies highlight the effect of peptide trapping inside (dead) cells on peptide activity and selectivity (19–21). It was shown that each cell can absorb ~10^7^ peptides (19–21). Often referred to as an *inoculum* effect [see (19–22) and references therein], this enhances population survivability (21), since it lowers the peptide concentration in the solution. As a result, the MIC obtained for a bacterial culture increases more rapidly with the cell density (21), compared to what corresponding model membranes would suggest (12, 13).

Here we offer a biophysical model of peptide activity and selectivity that assists with the correct interpretation of selectivity measurements. Our primary goal is to present a theoretical model, which can be used to predict peptide activity and selectivity under a variety of conditions, once their biophysical parameters are characterized. Indeed, an experimental approach to the relationship between peptide selectivity and cell densities is complex in a multi-species cultures, despite its relevance in biological and medical contexts. Our model will be beneficial for clarifying the relevance of selectivity measurements under controlled conditions.

Here we consider two approaches to quantifying cell selectivity (MHC/MIC). Imagine measuring MICs and MHCs in separate cell cultures (each containing a single species) and combining them into MHC/MIC. In this work, the resulting selectivity is referred to as “noncompetitive selectivity.” Alternatively, one can measure MICs and MHCs in a multi-species cell culture containing both bacteria and host cells and then calculate MHC/MIC. The resulting (competitive) selectivity is generally different from the corresponding noncompetitive one (12). If the competitive selectivity reflects adequately the competition between host cells and bacteria in binding peptides, the noncompetitive one can be exaggerated, when the host cell density is high, as correctly referred to as an experimental “illusion” by Matsuzaki (18).

Consistent with earlier studies (12, 13, 19–21), our results suggest that both MICs and MHCs increase with cell densities *C*_cell_; in a low cell-density limit, they become *C*_cell_-independent, i.e., intrinsic to a given peptide. Our results also show that peptide trapping increases both MICs and MHCs, magnifying their cell-density dependence, since the competition for peptides between cells is now stronger. This is a key feature highlighted in recent experiments (19–21) but left out in earlier theoretical studies (12, 13). The net effect of peptide trapping on peptide selectivity is that it tends to enhance the selectivity in the large host-cell density limit. With the parameter choices used, noncompetitive selectivity can be exaggerated by an order of magnitude. Our model also offers a systematic approach to correcting the selectivity for exaggeration; a noncompetitive selectivity can be corrected into a corresponding competitive one.

## Theoretical Model

In this section, we discuss how peptide selectivity depends on cell density. We first introduce a few key parameters relevant in this work. Let *C*_p_ be the total concentration of peptides. Recall that *P*/*L* is the molar ratio of membrane-bound peptides to lipids; (*P*/*L*)_B_ for bacterial membranes and (*P*/*L*)_H_ for host cell membranes. At a certain value of *C*_p_, denoted as $C_{\text{p}}^{*}$, *P*/*L* reaches a threshold value required for membrane rupture, (*P*/*L*)*; $C_{\text{p}}^{*}$ is either MIC or MHC. Also, the cell density, *C*_cell_, is a key parameter for peptide activity and selectivity (12, 13, 19–21); *C*_cell_ = *C*_B_ for bacteria and *C*_cell_ = *C*_H_ for host cells. A related quantity is the surface area of each cell, *A*_cell_ (12): *A*_cell_ = *A*_B_ or *A*_cell_ = *A*_H_ for bacteria and host cells, respectively. Doubling *A*_cell_ for given *C*_cell_ is equivalent to doubling *C*_cell_ for given *A*_cell_. Similarly, *a*_B_ and *a*_H_ are the lipid headgroup area for bacterial and host-cell membranes, respectively. Finally, *N*_p_ is the number of trapped peptides per cell: *N*_pB_ and *N*_pH_ for bacteria and host cells, respectively.

**Figure 1:**
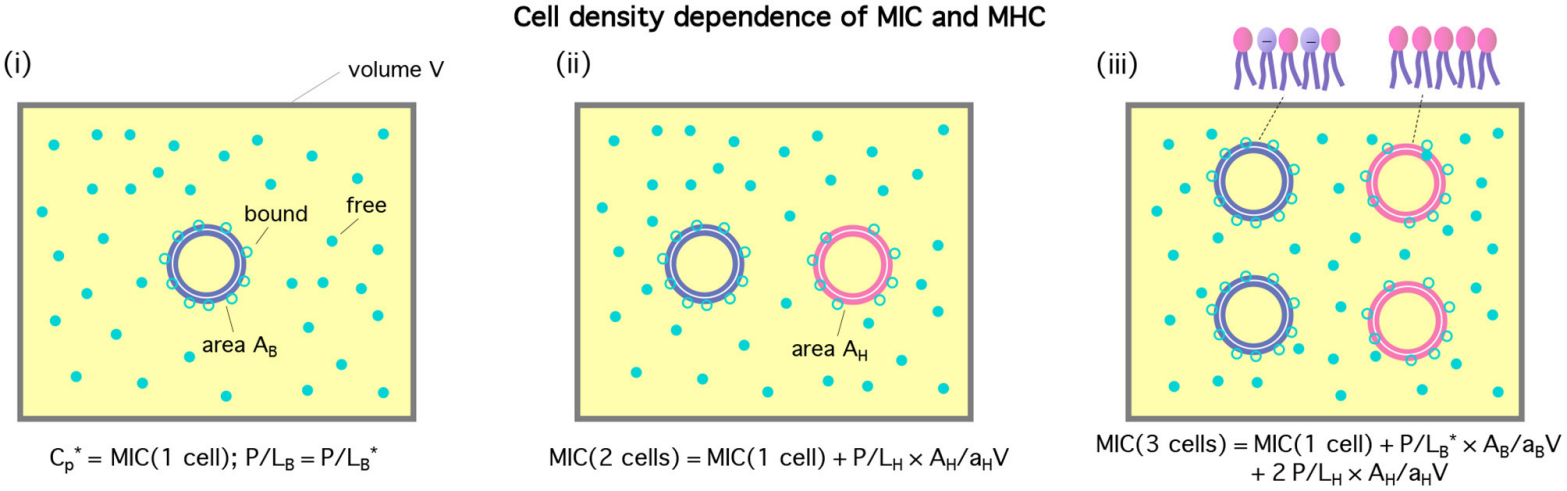
Cell-density dependence of $C_{\text{p}}^{*}$, i.e., either MIC or MHC. Cells are represented by two concentric circles and peptides by filled (free) or unfilled circles (bound) circles; if the blue circles represent bacterial cells, the pink ones stand for host cells. Let *A*_cell_ = *A*_B_ or *A*_H_ be the bacterial or host cell surface area, respectively. The progression from **(i)**–**(iii)** suggests that $\text{MIC}\left(C_{\text{cell}}\right)=\text{MI}{\text{C}}_{0}+A_{\text{B}}/a_{\text{B}}\times {\left(P/L\right)}_{\text{B}}^{*}C_{\text{B}}+A_{\text{H}}/a_{\text{H}}\times {\left(P/L\right)}_{\text{H}}C_{\text{H}}$. If we exchange the role between bacterial and host cells, we arrive at $\text{MHC}\left(C_{\text{cell}}\right)=\text{MH}{\text{C}}_{0}+A_{\text{H}}/a_{\text{H}}\times {\left(P/L\right)}_{\text{H}}^{*}C_{\text{H}}+A_{\text{B}}/a_{\text{B}}\times {\left(P/L\right)}_{\text{B}}C_{\text{B}}$. The figure was adapted with permission from (12), Copyright (2015) American Chemical Society, and from (13) with permission from The Royal Society of Chemistry.

The cell-density dependence of peptide activity, especially for a mixture of bacterial and host cells, is illustrated in Figure 1 [see (13) for a homogeneous case]. Here, the concentric circles in blue represent bacterial cells and the pink ones stand for host cells. Figure 1(i) shows a single-cell limit at an MIC. The introduction of a host cell will reduce the amount of peptides for the existing bacterial cell as shown in (ii). The extra number of peptides to maintain at the MIC is equal to (*P*/*L*)_H_ × *A*_H_/*a*_H_; similarly, in (iii), the number of peptides that should be added is ${\left(P/L\right)}_{\text{B}}^{*}\times A_{\text{B}}/a_{\text{B}}+2{\left(P/L\right)}_{\text{H}}\times A_{\text{H}}/a_{\text{H}}$.

A number of studies have unambiguously shown that (dead) cells can absorb a large number of peptides (~10^7^-10^8^) (19– 21). This enhances the so-called “inoculum” effect: it amplifies the cell-density dependence of MICs and MHCs, since it increases the number of peptides consumed by each cell. Along the line of what was done recently (13), this effect can be taken into account. Recall that *N*_pB_ and *N*_pH_ are the number of absorbed peptides per cell in bacterial and host cells, respectively. Our consideration replies on the following justifiable simplification: *N*_pB_ = 0 below MIC and similarly *N*_pH_ = 0 below MHC. Prior to membrane rupture, penetration of peptides into a cell is expected to be a rare event, since it involves overcoming a large free energy barrier for crossing an otherwise intact cell membrane.

Following the reasoning in Figure 1 and taking into account peptide trapping, one can arrive at


(1a) $$\begin{array}{l} \text{MIC}(C_{\text{B}},C_{\text{H}})={\text{MIC}}_{0}+\left[{\left(\frac{P}{L}\right)}_{\text{B}}^{*}\frac{A_{\text{B}}}{a_{\text{B}}}+N_{\text{pB}}^{*}\right]C_{\text{B}}\\ \;+{\left(\frac{P}{L}\right)}_{\text{H}}\frac{A_{\text{H}}}{a_{\text{H}}}C_{\text{H}} \end{array}$$

(1b) $$\begin{array}{l} \text{MHC}(C_{\text{B}},C_{\text{H}})={\text{MHC}}_{0}+\left[{\left(\frac{P}{L}\right)}_{\text{H}}^{*}\frac{A_{\text{H}}}{a_{\text{H}}}+N_{\text{pH}}^{*}\right]C_{\text{H}}\\ \;+\left[{\left(\frac{P}{L}\right)}_{\text{B}}\frac{A_{\text{B}}}{a_{\text{B}}}+N_{\text{pB}}\right]C_{\text{B}} \end{array}$$

Here MIC_0_ and MHC_0_ are, respectively, MIC and MHC in the low-cell density (or single-cell) limit: *C*_cell_ → 0 (*C*_cell_ is either *C*_B_ or *C*_H_). The term inside […] can be interpreted as the total number of peptides consumed per cell; recall $N_{\text{p}}^{*}$ is the value of *N*_p_ at $C_{\text{p}}^{*}$ (e.g., either MIC or MHC). It is assumed that MHC > MIC: peptides are selective, i.e., at the MIC, host cells remain intact. This has to be understood with caution. If MICs and MHCs are measured separately in a noncompetitive way, MICs can be larger than MHCs. This is, however, irrelevant for our discussion here. As a result of this inequality, the relations in Equation (1) are not fully symmetric with respect to the exchange between the subscripts “B” and “H.”

It is worth noting that the values of (*P*/*L*)_B_ and (*P*/*L*)_H_ depend on the total concentration of peptides and cell densities. They are determined by chemical equilibrium between free and bound peptides [see the Appendix]. In contrast, ${\left(P/L\right)}_{\text{B}}^{*}$ and ${\left(P/L\right)}_{\text{H}}^{*}$ are constants, which are set by the membrane-peptide parameters (3, 14–16).

Finally, note that the term [(*P*/*L*)_B_ (*A*_B_/*a*_B_) + *N*_pB_] in Equation (1b) is larger than […] in Equation (1a), since the former is evaluated at a larger value of *C*_p_ above the MIC. In this case, however, pore formation in bacterial membranes will alter the energetics of peptide binding. In the limit *C*_H_ ≫ *C*_B_, as is often the case, this will not limit the applicability of Equation (1b), since this term has a minimal impact on the MHC.

For a noncompetitive or homogeneous case, the last term in Equations (1a,b) will disappear. It is worth noting that the values of MIC_0_, MHC_0_, $N_{\text{pB}}^{*}$, and $N_{\text{pH}}^{*}$ can be obtained from noncompetitive measurements. If (*P*/*L*)* is not known, the number of peptides consumed per cell, i.e., the term inside […] in Equation (1), can be viewed as a fitting parameter. See below for a competitive case.

It will be instructive to compare the two terms inside […] in Equation (1): the number of membrane-bound peptides and the number of adsorbed peptides per cell. For this consideration, we invoke some simplification: a cell viewed as a sack of molecules enclosed by a bilayer. For *E. coli* as a representative bacterium, $A_{\text{B}}\approx 12\text{μ}{\text{m}}^{2}$, twice the area of each lipid layer (either inner or outer) in the cytoplasmic membrane. Since $a_{\text{B}}\approx a_{\text{H}}\approx 70{Å}^{2}$, $A_{\text{B}}/a_{\text{B}}\approx 1.7\times 10^{7}$. For the peptide melittin, ${\left(P/L\right)}_{\text{B}}^{*}\approx 0.02$ and ${\left(P/L\right)}_{\text{H}}^{*}\approx 0.01$ (14–16). We thus find ${\left(P/L\right)}_{\text{B}}^{*}\left(A_{\text{B}}/a_{\text{B}}\right)\approx 3.4\times 10^{5}$. This number is much smaller than $N_{\text{pB}}\approx 10^{7}$-10^8^ (21). The presence of outer membranes will not change this inequality. For human red blood cells as representative host cells, *A*_H_ ≈ 17*A*_B_ and $A_{\text{H}}/a_{\text{H}}\approx 2.9\times 10^{8}$. As a result, we obtain ${\left(P/L\right)}_{\text{H}}^{*}\left(A_{\text{H}}/a_{\text{H}}\right)\approx 2.9\times 10^{6}$, which is smaller than $N_{\text{pH}}\approx 10^{7}$ (19, 20). The main source of inoculum effects is the trapping of peptides inside dead cells (i.e., for *P*/*L* > (*P*/*L*)*).

A full analysis of Equation (1) is involved, since it requires the determination of four unknowns: (*P*/*L*)_B_, (*P*/*L*)_H_, *N*_pB_, and *N*_pH_, as a function of *C*_p_ [see (12, 13) for earlier efforts]; also the energetics of peptide trapping including peptide binding to intracellular components has yet to be understood in a quantitative manner.

In some relevant limits, we can use Equation (1) to map out a few scenarios regarding peptide selectivity. In the competitive case, if *C*_H_ ≫ *C*_B_ as in whole blood, Equation (1) can be approximated as


(2a) $$\text{MIC}(C_{\text{B}},C_{\text{H}})\approx {\text{MIC}}_{0}+{\left(\frac{P}{L}\right)}_{\text{H}}\frac{A_{\text{H}}}{a_{\text{H}}}C_{\text{H}},$$

(2b) $$\text{MHC}(C_{\text{B}},C_{\text{H}})\approx {\text{MHC}}_{0}+\left[{\left(\frac{P}{L}\right)}_{\text{H}}^{*}\frac{A_{\text{H}}}{a_{\text{H}}}+N_{\text{pH}}^{*}\right]C_{\text{H}}$$

Here (*P*/*L*)_H_ in Equation (2a) is to be evaluated at *C*_p_ = MIC.

In Equation (2), MIC_0_ and MHC_0_ can be viewed as fitting parameters. In a more systematic approach, they can be related to binding energy, *w*, which characterizes the interaction of a peptide with a membrane (see the Appendix); in this work, *w*_B_ and *w*_H_ are the binding energy for bacterial and host-cell membranes, respectively.

Chemical equilibrium between free and bound peptides [see Equation A3 in the Appendix and the SI of (12)] leads to^1^


(3) $$\text{MIC}(C_{\text{B}},C_{\text{H}})\approx \frac{1}{v_{\text{p}}}\cdot \frac{\frac{A_{\text{p}}}{a_{\text{H}}}{\left(\frac{P}{L}\right)}_{\text{H}}}{1-\frac{A_{\text{p}}}{a_{\text{H}}}{\left(\frac{P}{L}\right)}_{\text{H}}}e^{w_{\text{H}}/k_{\text{B}}T}+{\left(\frac{P}{L}\right)}_{\text{H}}\frac{A_{\text{H}}}{a_{\text{H}}}C_{\text{H}}.$$

Here, *v*_p_ is the volume occupied by each peptide in the bulk and *A*_p_ is the peptide area on the membrane surface.

We can use Equation (3) to eliminate (*P*/*L*)_H_ in Equation (2a) by equating the first terms in these two equation^2^; similarly, ${\left(P/L\right)}_{\text{H}}^{*}$ in Equation (2b) can be eliminated in favor of (MHC)_0_:


(4a) $$\text{MIC}(C_{\text{B}},C_{\text{H}})\approx {\text{MIC}}_{0}+\left(\frac{{\text{MIC}}_{0}v_{\text{p}}}{{\text{MIC}}_{0}v_{\text{p}}+e^{w_{\text{H}}/k_{\text{B}}T}}\frac{A_{\text{H}}}{A_{\text{p}}}\right)C_{\text{H}},$$

(4b) $$\begin{array}{l} \text{MHC}(C_{\text{B}},C_{\text{H}})\approx {\text{MHC}}_{0}\\ \;+\left(\frac{{\text{MHC}}_{0}v_{\text{p}}}{{\text{MHC}}_{0}v_{\text{p}}+e^{w_{\text{H}}/k_{\text{B}}T}}\frac{A_{\text{H}}}{A_{\text{p}}}+N_{\text{pH}}^{*}\right)C_{\text{H}}. \end{array}$$

The MIC in Equation (4a) increases linearly with *C*_H_. It can be strikingly different from the corresponding noncompetitive MIC in the limit *C*_B_ → 0: MIC_0_. For sufficiently large *C*_H_, the former can be much larger than the latter.

The ratio MHC/MIC becomes


(5) $$\begin{array}{l} \frac{\text{MHC}}{\text{MIC}}\approx \frac{{\text{MHC}}_{0}+\left[{\left(\frac{P}{L}\right)}_{\text{H}}^{*}\frac{A_{\text{H}}}{a_{\text{H}}}+N_{\text{pH}}^{*}\right]C_{\text{H}}}{{\text{MIC}}_{0}+\left[\frac{{\text{MIC}}_{0}v_{\text{p}}}{{\text{MIC}}_{0}v_{\text{p}}+e^{w_{\text{H}}/k_{\text{B}}T}}\frac{A_{\text{H}}}{A_{\text{p}}}\right]C_{\text{H}}}\\ \;=\frac{{\text{MHC}}_{0}+\left(\frac{{\text{MHC}}_{0}v_{\text{p}}}{{\text{MHC}}_{0}v_{\text{p}}+e^{w_{\text{H}}/k_{\text{B}}T}}\frac{A_{\text{H}}}{A_{\text{p}}}+N_{\text{pH}}^{*}\right)C_{\text{H}}}{{\text{MIC}}_{0}+\left(\frac{{\text{MIC}}_{0}v_{\text{p}}}{{\text{MIC}}_{0}v_{\text{p}}+e^{w_{\text{H}}/k_{\text{B}}T}}\frac{A_{\text{H}}}{A_{\text{p}}}\right)C_{\text{H}}}. \end{array}$$

This implies that peptide trapping in host cells enhances peptide selectivity. Compared to the case $N_{\text{pH}}^{*}\approx 0$, more peptides will be needed in order for (*P*/*L*)_H_ to reach ${\left(P/L\right)}_{\text{H}}^{*}$ for $N_{\text{pH}}^{*}\gg 1$. Since the second term inside […] in the numerator of Equation (5) is larger than the first term roughly by an order of magnitude, the effect of peptide trapping on the selectivity is up to about 10-fold.

Note that the MHC in Equation (4b) holds for a host-cell only case as well. In contrast, the MIC for a bacterial-cell only case becomes


(6) $$\text{MIC}(C_{\text{B}})={\text{MIC}}_{0}+\left(\frac{{\text{MIC}}_{0}v_{\text{p}}}{{\text{MIC}}_{0}v_{\text{p}}+e^{w_{\text{B}}/k_{\text{B}}T}}\frac{A_{\text{B}}}{A_{\text{p}}}+N_{\text{pB}}^{*}\right)C_{\text{B}}.$$

This can be obtained from Equation (4b) by exchanging the role of host cells with that of bacteria.

The main advantage of Equations (4), (5), and (6) is that *P*/L* is not shown explicitly. It is absorbed into MIC_0_ or MHC_0_, which are experimentally more accessible. Also it is worth noting that the use of Equation (4) or Equation (5) would not necessarily require measurements of such biophysical parameters as *v*_p_, *w*_H_, *w*_B_, $N_{\text{pH}}^{*}$, and $N_{\text{pB}}^{*}$. The term inside (…) on the right-hand side of Equations (4) and (6) as a whole can be viewed as a fitting parameter. It is a slope of either MIC or MHC curve as a function of the cell density and can be obtained from the corresponding homogeneous case. See the last section for relevant points.

## Results

We have analyzed Equations (4) and (5) to clarify inoculum effects on peptide activity and selectivity. For lipid bilayers mimicking cell membranes, the parameters in these equations have been characterized (12–16). They are, however, not known for real cells. In particular, the interdependence between *w*, *P*/L*, and $C_{\text{p}}^{*}$ is elusive because of the complexities of cell structures. For instance, *w*_B_ for Gram-negative bacteria should take into account the peptide interaction with their outer membrane (OM), among others. Recall that this is an effective parameter, in which microscopic details (e.g., peptide charge, peptide interaction with the OM, and the presence of cholesterol in the host-cell membrane) are subsumed. This has only recently been mapped out theoretically for lipid bilayers (13). Here we do not attempt to calculate the effective binding energy *w* (either *w*_B_ or *w*_H_) for real cells and to use it in the computation of MIC_0_ and MHC_0_. Instead, we start with conveniently-chosen but biophysically-relevant values of MIC_0_ and MHC_0_. The resulting analysis will not involve (*P*/*L*)* explicitly. For simplicity, the number of trapped peptides *N*_p_ is chosen to be the same for bacteria and host cells: $N_{\text{p}}=0,10^{7},5\times 10^{7}$.

Otherwise, we have used peptide parameters relevant for the peptide melittin (12–16): peptide charge *Q* = 5, $A_{\text{p}}=400{Å}^{2}$, and $v_{\text{p}}=33^{3}{Å}^{3}$. For this peptide, *w* was mapped out for model membranes, mimicking bacterial and host-cell membranes: *w*_B_ = −16.6*k*_B_*T* and *w*_H_ = −6.72*k*_B_*T* (13). They are used as representative binding energy. Also, $a_{\text{B}}=74{Å}^{2}$, $a_{\text{H}}=71{Å}^{2}$, $A_{\text{B}}=1.2\times 10^{9}{Å}^{2}=12\mu {\text{m}}^{2}$ as for *E. coli*, and *A*_H_ = *A*_B_ or *A*_H_ = 17*A*_B_ as for human red blood cells (12).

**Figure 2:**
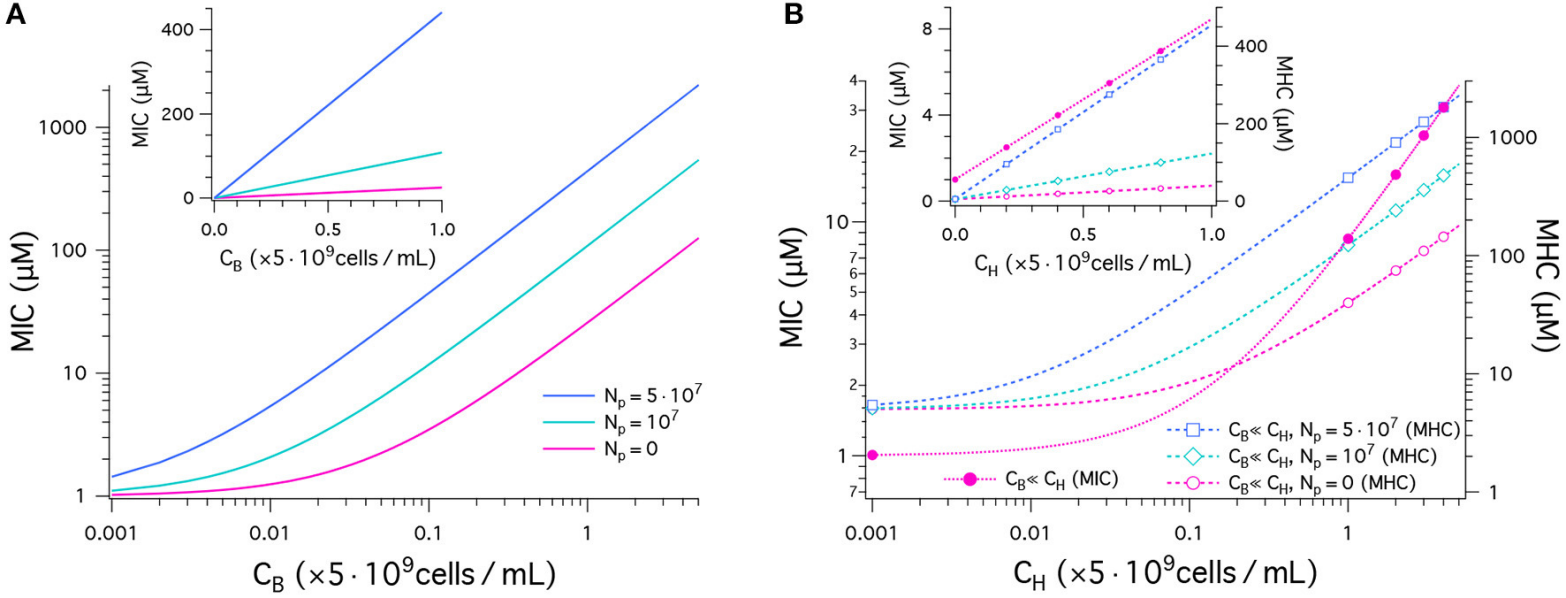
Peptide activity, i.e., MICs and MHCs. We have chosen the parameters as follows: (MIC)_0_ = 1μM and (MHC)_0_ = 5μM; *w*_B_ = −16.6*k*_B_*T* and *w*_H_ = −6.72*k*_B_*T*; $A_{\text{p}}=400^{2}$; $v_{\text{p}}=33^{3\text{ 3}}$; $A_{\text{B}}=1.2\times 10^{9\text{ 2}}$ and *A*_H_ = 17 × *A*_B_. The number of trapped peptides *N*_p_ is chosen to be the same for bacteria and host cells: $N_{\text{p}}=0,10^{7},5\times 10^{7}$. **(A)** This graph shows the results for MICs as a function of *C*_B_ in units of 5 × 10^9^cells/mL obtained in a noncompetitive way. In all cases, the MIC increases from MIC_0_ = 1, as *C*_B_ increases (Equation 1). The MIC is higher for a larger value of *N*_p_. The sensitivity of the MIC to *N*_p_ is better captured in the linear plot in the inset; all the curves indicate a linear relationship between the MIC and *C*_B_. **(B)** MICs (left axis) and MHCs (right axis) are shown as a function of *C*_H_ given in units of 5 × 10^9^cells/mL obtained in a competitive way. Various symbols are used to distinguish between different choices of *N*_p_. If *C*_H_ ≫ *C*_B_, MICs are roughly independent of *N*_p_; in this case, MHCs are approximately the same for the competitive and noncompetitive cases. As *C*_H_ increases, the MIC increases up to 40-fold from MIC_0_ at *C*_H_ = 0 (Equation 4a). Similarly MHCs increase as a function of *C*_H_, more rapidly for larger *N*_p_ (Equation 4b); for $N_{\text{p}}=10^{7}$, the MHC increases by up to two orders of magnitude. The inset graph recaptures the data in a linear plot.

We have plotted our results for MICs and MHCs in Figure 2. For this, we have chosen the parameters as follows: MIC_0_ = 1μM and MHC_0_ = 5μM. Figure 2A shows the MIC as a function of *C*_B_ in units of 5 × 10^9^cells/mL obtained in a noncompetitive way. In all cases, the MIC increases linearly from MIC_0_ = 1μM, as *C*_B_ increases, as expected from Equation (1). The inset recaptures the MIC data in linear plot. It indicates a linear relationship between the MIC and *C*_B_. The MIC curve is steeper for a larger value of *N*_p_. This is well aligned with recent experiments (21). The inoculum effect increases the slope of the MIC curves, not the “*y*”-intercept, which coincides with cell-density independent MIC_0_.

In Figure 2B, MICs (left axis) and MHCs (right axis) are shown as a function of *C*_H_ given in units of 5 × 10^9^cells/mL obtained in a competitive way. They are represent by dashed lines with symbols. First, note that MHCs are approximately the same for the competitive and noncompetitive cases as long as *C*_H_ ≫ *C*_B_; also MICs are insensitive to *C*_B_ and *N*_p_, if *C*_H_ ≫ *C*_B_ and MHC_0_ > MIC_0_ (see Equation 4). This is distinct from larger MICs for larger *N*_p_ in the noncompetitive case in Figure 2A. As *C*_H_ increases, the MIC increases up to 40-fold from MIC_0_ at *C*_H_ = 0 (Equation 4A). This is consistent with the observation that peptide interactions with host cells diminish peptide activity *in vivo* (24). Similarly, MHCs increase as a function of *C*_H_, more rapidly for larger *N*_p_ (Equation 4B and the inset graph). For $N_{\text{p}}=10^{7}$, the MHC increases by up to two orders of magnitude.

**Figure 3:**
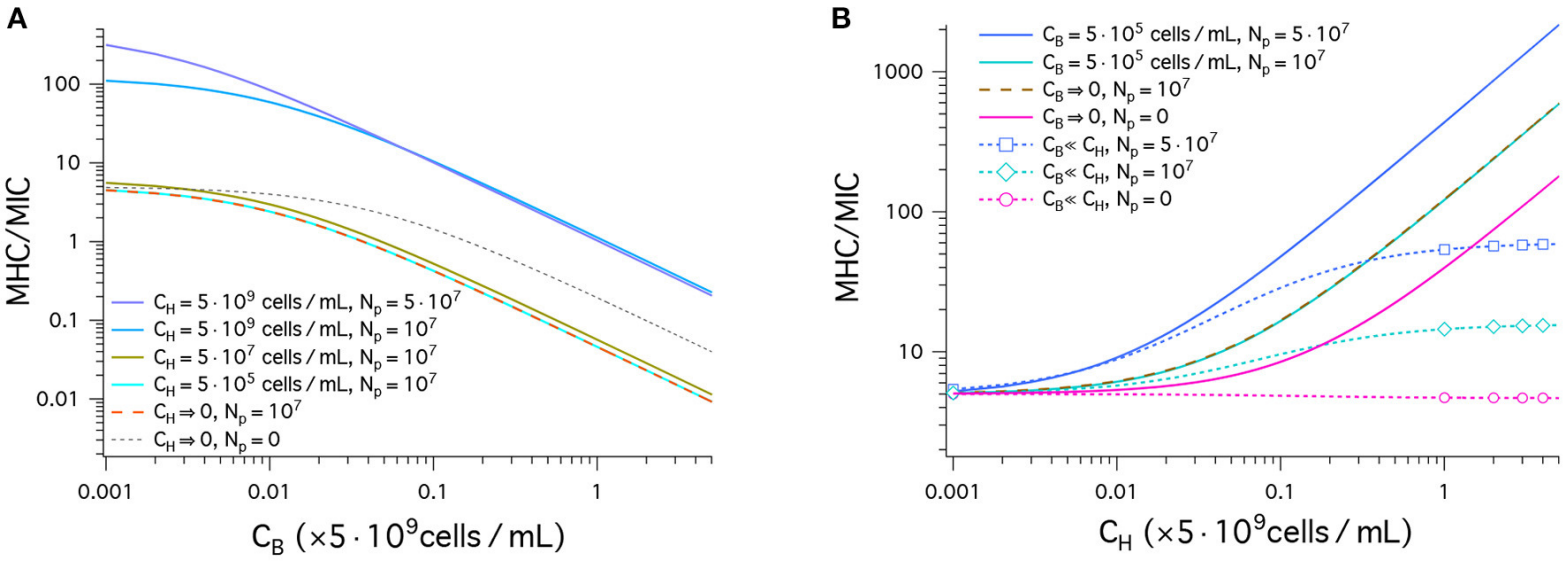
Cell selectivity of antimicrobial peptides, i.e., MHC/MIC. We have used the same parameters as in Figure 2: (MIC)_0_ = 1μM, and (MHC)_0_ = 5μM; *w*_B_ = −16.6*k*_B_*T*, and *w*_H_ = −6.72*k*_B_*T*; $A_{\text{p}}=400^{2}$; $v_{\text{p}}=33^{3\text{ 3}}$; $A_{\text{B}}=1.2\times 10^{9\text{ 2}}$ and *A*_H_ = 17 × *A*_B_; $N_{\text{p}}=0,10^{7},5\times 10^{7}$ (the same for bacteria and host cells). **(A)** This graph shows MHC/MIC as a function of *C*_B_ in units of 5 × 10^9^cells/mL obtained in a noncompetitive way. In all cases, the selectivity decreases, as *C*_B_ increases. The selectivity is higher for larger values of *C*_H_. It is also larger for larger *N*_p_ unless *C*_H_ = 0 (black dashed) or $C_{\text{B}}≳0.07\times 5\times 10^{9}$ (compare the top two curves). Also note that there is no essential difference between the two cases: $C_{\text{H}}=0,N_{\text{p}}=10^{7}$ (tangerine) and $C_{\text{H}}=5\times 10^{5}\text{cells}/\text{mL},N_{\text{p}}=10^{7}$ (cyan). This means that the latter case falls in the single-cell limit. **(B)** MHC/MIC are shown as a function of *C*_H_ given in units of 5 × 10^9^cells/mL. Competitive (dashed lines with various symbols) and noncompetitive (solid lines) cases are compared. For the competitive case, Equation (4) was used, which holds for *C*_H_ ≫ *C*_B_. The competitive selectivity increases as *C*_H_ increases, except for *N*_p_ = 0 (magenta). In all noncompetitive cases shown, the selectivity increases as *C*_H_ increases. In all cases, the selectivity is higher for larger *N*_p_. In the noncompetitive case, the presence of 5 × 10^5^cells/mL does not change the selectivity with reference to the corresponding limiting case *C*_B_ → 0; at this density of bacterial cells, MIC ≈ MIC_0_. Compared to the corresponding competitive selectivity, the noncompetitive selectivity is overestimated, more so for larger *C*_H_; for $C_{\text{H}}=5\times 10^{9}\text{cells}/\text{mL}$, the latter is exaggerated by an order of magnitude.

Figure 3 displays our results for peptide selectivity, which combines the graphs in Figures 2A and B. The graph in Figure 3A shows our results for MHC/MIC as a function of *C*_B_ obtained in a noncompetitive way. In all cases presented by various colors, the ratio MHC/MIC or the selectivity decreases, as *C*_B_ increases. The selectivity is higher for larger values of *C*_H_. Also, it is higher for larger *N*_p_ if $C_{\text{B}}≲0.07\times 10\times 10^{9}\text{cells}/\text{mL}$ but is smaller if $C_{\text{B}}≳0.07\times 10\times 10^{9}\text{cells}/\text{mL}$. Peptide trapping increases both MHC and MIC. At low *C*_B_, the net effect is to enhance the selectivity; at high *C*_B_, it reduces the selectivity, since lots of peptides are trapped in bacteria and “wasted.”

Also note that there is no essential difference between the two cases: $C_{\text{H}}=0,N_{\text{p}}=10^{7}$ (tangerine) and $C_{\text{H}}=5\times 10^{5}\text{cells}/\text{mL},N_{\text{p}}=10^{7}$ (cyan). This means that the latter case falls in the single-cell limit.

In Figure 3B, the results for MHC/MIC are shown as a function of *C*_H_. Competitive (dashed line with various symbols) and noncompetitive (solid lines) cases are compared. For the competitive case, Equation (4) was used, which holds for *C*_H_ ≫ *C*_B_. The competitive selectivity increases as *C*_H_ increases, except for *N*_p_ = 0 (magenta). In all noncompetitive cases, the selectivity increases as *C*_H_ increases; the presence of $C_{\text{B}}=5\times 10^{5}\text{cells}/\text{mL}$ does not change the selectivity with reference to the corresponding limiting case *C*_B_ → 0, since at this density of bacterial cells, MIC ≈ MIC_0_. In both the competitive and noncompetitive cases shown, the selectivity is higher for larger *N*_p_: peptide trapping enhances the selectivity.

Similarly to what earlier studies suggest (12, 18), the results in Figure 3B show how peptide selectivity can be mistakenly estimated. Compared to the corresponding competitive selectivity, the noncompetitive selectivity is overestimated, more so for larger *C*_H_; for $C_{\text{H}}=5\times 10^{9}\text{cells}/\text{mL}$, the latter is exaggerated by an order of magnitude.

These results also clear up possible confusions. Even in the presence of a large amount of host cells, the selectivity measured in a competitive environment is not an experimental artifact. It just reflects correctly the cell-density dependence of the selectivity, as discussed in the section 2.

## Discussions and Conclusions

We have discussed the cell-density dependence of peptide activity and selectivity. For this, we have combined physical arguments, which relate peptide activity and selectivity to cell density, and a Langmuir-type model, in which the amount peptide binding (or trapping) is dictated by an effective binding energy. This combined effort produced a predictive model for peptide activity and selectivity. It can be used to calculate MICs, MHCs, and MHC/MIC, once a few key biophysical parameters are characterized, which include the number of trapped peptides per cell (19–21) and peptide-membrane interactions.

Alternatively, our model can be used as a fitting model for analyzing data. For instance, the “*y*”-intercept and the “slope” can be extracted from noncompetitive measurements of MICs or MHCs vs. cell density. This will determine (MIC)_0_ or (MHC)_0_ as well as the terms inside (…) on the right-hand side of Equations (4b) and (6). This information can be used in Equation (4) (or more generally Equation 1), which represents a heterogeneous mixture of bacteria and host cells.

This consideration, however, would necessitate prior knowledge about one of $N_{\text{pB}}^{*}$ and ${\left(P/L\right)}_{\text{B}}^{*}$ (or equivalently *w*_B_). To see this, notice that homogeneous measurements lead to the value of the sum of the two terms inside (…) in Equation (6). If ${\left(P/L\right)}_{\text{B}}^{*}$ is known, as is most obvious for pure-lipid membranes (3, 17), $N_{\text{pB}}^{*}$ can be extracted from noncompetitive measurements.

An alternative but possibly less practical approach is to measure several MICs in a competitive setting. By fitting the data to Equation (4a) will produce the coefficient of *C*_H_. One can then obtain MIC, MHC, and MHC/MIC as a function of *C*_B_ or *C*_H_, the density of bacteria or host cells, respectively. For instance, in whole blood, $C_{\text{H}}\approx 5\times 10^{9}\text{cells}/\text{mL}$. The density of bacteria depends on the degree and location of infection. It ranges from 1 colony-forming unit (CFU/mL) (in blood stream) to 10^9^CFU/mL (in soft tissue or peritonea) [see a recent review (20) and relevant references therein]. Graphs similar to those in Figure 2 or Figure 3 will be beneficial for understanding the activity and selectivity of antimicrobial peptides in varying biological environments.

As pointed out in a number of earlier studies (12, 18–20), the selectivity measured noncompetitively is often much larger than the corresponding competitive one, if the host cell density is much larger than the bacterial cell density. The results in Figure 3 offer a quantitative picture of how the selectivity can be excessively overestimated. It can, however, be corrected, since noncompetitive measurements can be converted into competitive ones. For instance, suppose that noncompetitive measurements led to *w*_B_ = −16.6*k*_B_*T*, *w*_H_ = −6.72*k*_B_*T*, $N_{\text{p}}=10^{7}$, MIC_0_ = 1μM, and MHC_0_ = 5μM, as in Figure 3. In the presence of $C_{\text{B}}=5\times 10^{5}\text{cells}/\text{mL}$ and $C_{\text{H}}=5\times 10^{9}\text{cells}/\text{mL}$ (*C*_B_ ≪ *C*_H_), these parameters choices would lead to the following noncompetitive selectivity: MHC/MIC ≈ 100 (Equation 1 and Figure 3). It can be corrected graphically (Figure 3) or mathematically (Equation 4) into the corresponding competitive selectivity MHC/MIC ≈ 10.

As a final remark, we wish to mention that peptide activity against live cells is time-dependent, as observed in recent experiments (21). Accordingly, the density of bacterial cells, is a dynamic quantity. Furthermore, heterogeneous absorption of peptides in cells was shown to have a nontrivial consequence on population survivability. Because of the stochastic nature of molecular interactions occurring on the cell surface and inside, some cells absorb a large number of peptides (~10^7^-10^8^) (19–21), thus reducing the availability of peptides to the rest and contributing favorably to population survivability (21). Also, the density of peptides can change with time, depending on how fast the host cells produce them (21). It is also influenced by peptide degradation by protease (20, 24). Its effect on peptide activity is similar to what we expect from peptide trapping. Taking into all these known and unknown details goes beyond the scope of what can be done at present. Future considerations are warranted.

## Data Availability Statement

The raw data supporting the conclusions of this article will be made available by the authors, without undue reservation.

## Author Contributions

B-YH and BRS conducted the research. B-YH wrote the manuscript. BRS, SN, and ST-A commented on the manuscript. SN helped solve the peptide binding equations. All authors contributed to the article and approved the submitted version.

## Conflict of Interest

The authors declare that the research was conducted in the absence of any commercial or financial relationships that could be construed as a potential conflict of interest.

## Footnotes

^1^Here (*P*/*L*)_H_ is to be evaluated at the peptide concentration *C*_p_ = MIC. As a result, *w*_H_ in this expression corresponds to (*P*/*L*)_H_ smaller than ${\left(P/L\right)}_{\text{H}}^{*}$. Here we ignore the possible dependence of *w*_H_ on (*P*/*L*)_H_. For pure-lipid membranes, this dependence can, in principle, be mapped out (13). If we use the final expression in Equation (4a) as a fitting model, this issue becomes irrelevant.

^2^The origin of the cell-density dependent term in Equation (3) is obvious from the illustration in Figure 1. At the low-cell density limit, Equation (3) is equivalent to saying that

(7) $${\text{MIC}}_{0}=\frac{1}{v_{\text{p}}}\cdot \frac{\frac{A_{\text{p}}}{a_{\text{H}}}{\left(\frac{P}{L}\right)}_{\text{H}}}{1-\frac{A_{\text{p}}}{a_{\text{H}}}{\left(\frac{P}{L}\right)}_{\text{H}}}e^{w_{\text{H}}/k_{\text{B}}T}$$

This can be obtained from Equation (A3) in the Appendix. More directly, chemical equilibrium at *C*_p_ = MIC_0_ in the low-cell density limit requires

(8) $$\begin{array}{l} \ln\left(v_{\text{p}}{\text{MIC}}_{0}\right)=\frac{w_{\text{B}}}{k_{\text{B}}T}+\ln\frac{\frac{A_{\text{p}}}{a_{\text{B}}}{\left(\frac{P}{L}\right)}_{\text{B}}^{*}}{1-\frac{A_{\text{p}}}{a_{\text{B}}}{\left(\frac{P}{L}\right)}_{\text{B}}^{*}}\\ \;=\frac{w_{\text{H}}}{k_{\text{B}}T}+\ln\frac{\frac{A_{\text{p}}}{a_{\text{H}}}{\left(\frac{P}{L}\right)}_{\text{H}}}{1-\frac{A_{\text{p}}}{a_{\text{H}}}{\left(\frac{P}{L}\right)}_{\text{H}}}. \end{array}$$

The second term in each line is the entropic chemical potential of bound peptides in units of *k*_B_*T* (23). The second equality leads to Equation (7), which shows the relationship between the total peptide concentration, i.e., MIC_0_, and (*P*/*L*)_H_. Equation (7) can readily be solved for (*P*/*L*)_H_:

(9) $${\left(\frac{P}{L}\right)}_{\text{H}}=\frac{{\text{MHC}}_{0}v_{\text{p}}}{{\text{MHC}}_{0}v_{\text{p}}+e^{w_{\text{H}}/k_{\text{B}}T}}.$$

This is used in the transition from Equations (2A) to (4A).

## Appendix

Here we present a Langmuir model of peptide binding [see (23) and the SI of (12)]. Let μ_B_ and μ_H_ be the chemical potential of bound peptides, and σ_B_ and σ_H_ their planar density, on the bacterial and host-cell surface, respectively. The planar density is related to *P*/*L* through σ_B_*a*_B_ = (*P*/*L*)_B_ and σ_H_*a*_H_ = (*P*/*L*)_H_. Recall that *w*_B_ and *w*_H_ are the peptide binding energy for bacterial and host-cell membranes, respectively. In general, the binding energy depends on the value of *P*/*L* mainly through the interaction between bound peptides. In recent studies (13), it was estimated at *P*/*L* = (*P*/*L*)*. The resulting binding energy can be used to find (*P*/*L*)*, i.e., either MIC or MHC.

Let *v*_p_ be the volume occupied by each peptide in the bulk and *A*_p_ the area occupied by each bound peptide on the membrane surface. In the presence of two types of cells, we find

(A1)
$$\begin{array}{l} \mu_{\text{B}}=w_{\text{B}}+k_{\text{B}}T\ln\left(\frac{\sigma_{\text{B}}A_{\text{p}}}{1-\sigma_{\text{B}}A_{\text{p}}}\right)\\ \mu_{\text{H}}=w_{\text{H}}+k_{\text{B}}T\ln\left(\frac{\sigma_{\text{H}}A_{\text{p}}}{1-\sigma_{\text{H}}A_{\text{p}}}\right) \end{array}$$

as well as

(A2)
$$\mu_{\text{free}}=k_{\text{B}}T\ln\{\left[C_{\text{p}}-\left(C_{\text{B}}\sigma_{\text{B}}A_{\text{B}}+C_{\text{H}}\sigma_{\text{H}}A_{\text{H}}\right)\right]v_{\text{p}}\}.$$

In equilibrium, μ_B_ = μ_H_ = μ_free_. We thus arrive at

(A3a)
$$C_{\text{p}}={\left(\frac{P}{L}\right)}_{\text{B}}\frac{A_{\text{B}}}{a_{\text{B}}}C_{\text{B}}+{\left(\frac{P}{L}\right)}_{\text{H}}\frac{A_{\text{H}}}{a_{\text{H}}}C_{\text{H}}+\frac{1}{v_{\text{p}}}\frac{\frac{A_{\text{p}}}{a_{\text{B}}}{\left(\frac{P}{L}\right)}_{\text{B}}}{1-\frac{A_{\text{p}}}{a_{\text{B}}}{\left(\frac{P}{L}\right)}_{\text{B}}}e^{w_{\text{B}}/k_{\text{B}}T}$$

(A3b)
$$C_{\text{p}}={\left(\frac{P}{L}\right)}_{\text{B}}\frac{A_{\text{B}}}{a_{\text{B}}}C_{\text{B}}+{\left(\frac{P}{L}\right)}_{\text{H}}\frac{A_{\text{H}}}{a_{\text{H}}}C_{\text{H}}+\frac{1}{v_{\text{p}}}\frac{\frac{A_{\text{p}}}{a_{\text{H}}}{\left(\frac{P}{L}\right)}_{\text{H}}}{1-\frac{A_{\text{p}}}{a_{\text{H}}}{\left(\frac{P}{L}\right)}_{\text{H}}}e^{w_{\text{H}}/k_{\text{B}}T}.$$

In this expression, we eliminated the planar density in favor of *P*/*L*. These equations can be solved simultaneously for the two unknowns: (*P*/*L*)_H_ and (*P*/*L*)_B_ for a given value of *C*_p_. The value of *C*_p_ at which ${\left(P/L\right)}_{\text{H}}={\left(P/L\right)}_{\text{H}}^{*}$ (${\left(P/L\right)}_{\text{B}}={\left(P/L\right)}_{\text{B}}^{*})$ is an MHC (MIC). If evaluated at *P*/*L**, the last term in Equations A3(a) and (b) is the $C_{\text{p}}^{*}$ in the low-cell density limit: either MHC_0_ or MIC_0_.
